# Branched-chain and aromatic amino acid profiles and diabetes risk in Chinese populations

**DOI:** 10.1038/srep20594

**Published:** 2016-02-05

**Authors:** Tianlu Chen, Yan Ni, Xiaojing Ma, Yuqian Bao, Jiajian Liu, Fengjie Huang, Cheng Hu, Guoxiang Xie, Aihua Zhao, Weiping Jia, Wei Jia

**Affiliations:** 1 Shanghai Key Laboratory of Diabetes Mellitus and Center for Translational Medicine, Shanghai Jiao Tong University Affiliated Sixth People’s Hospital, Shanghai 200233, China; 2 University of Hawaii Cancer Center, Honolulu, HI 96813, USA; 3 Department of Endocrinology and Metabolism, Shanghai Jiao Tong University Affiliated Sixth People’s Hospital; Shanghai Diabetes Institute; Shanghai, 200233, China

## Abstract

Recent studies revealed strong evidence that branched-chain and aromatic amino acids (BCAAs and AAAs) are closely associated with the risk of developing type 2 diabetes in several Western countries. The aim of this study was to evaluate the potential role of BCAAs and AAAs in predicting the diabetes development in Chinese populations. The serum levels of valine, leucine, isoleucine, tyrosine, and phenylalanine were measured in a longitudinal and a cross sectional studies with a total of 429 Chinese participants at different stages of diabetes development, using an ultra-performance liquid chromatography triple quadruple mass spectrometry platform. The alterations of the five AAs in Chinese populations are well in accordance with previous reports. Early elevation of the five AAs and their combined score was closely associated with future development of diabetes, suggesting an important role of these metabolites as early markers of diabetes. On the other hand, the five AAs were not as good as existing clinical markers in differentiating diabetic patients from their healthy counterparts. Our findings verified the close correlation of BCAAs and AAAs with insulin resistance and future development of diabetes in Chinese populations and highlighted the predictive value of these markers for future development of diabetes.

The prevalence of obesity and metabolic syndrome (MS) have reached epidemic proportions^1^. Obesity and the MS are strongly linked to the development of diabetes, hypertension, cardiovascular disease, coronary heart disease, and several types of cancers^2^,^3^. Therefore, the identification of individuals at risk of developing metabolic diseases before the MS is of particular importance for prevalence control and early intervention.

A number of studies have reported that the serum levels of branched-chain and aromatic amino acids (BCAAs and AAAs), including leucine, isoleucine, valine, phenylalanine, and tyrosine, are significantly different among lean, obesity, and diabetes, and are closely correlated to insulin resistance, highlighting their potential for diabetes diagnosis and risk assessment^4^,^5^,^6^,^7^. Recently, the significant associations of the five amino acids (AAs) with insulin resistance, obesity, and future diabetes were identified and verified in American individuals^8^,^9^,^10^ and young Finns^11^. The mechanistic linkage between these five AAs and insulin resistance were investigated by Langenberg *et al.*^12^ and Newguard^13^, respectively. More recently, Tilin *et al.* reported ethnical differences in the blood levels of these amino acids and suggested that these differences may add explanatory insights into the increased risk of diabetes in South Asian populations compared with Europeans^14^. However, these cohort studies did not involve Chinese populations. It has been well documented that most diabetic patients in China have a lower BMI and impaired islet function at the early stages of metabolic diseases^15^, necessitating independent Chinese population studies on these amino acid markers. Our group recently reported a gender difference in blood metabolite profiles including BCAAs and AAAs between obese men and women in China^16^, suggesting that the risk prediction ability of these amino acid markers may be gender dependent.

The main goal of this study was to evaluate the five AAs in predicting the risk of developing diabetes in Chinese populations. Using a mass spectrometry platform, we measured the levels of leucine, isoleucine, valine, phenylalanine, and tyrosine in 429 serum samples from two independent groups of individuals (supplemental Fig. 1). Specifically, we examined the baseline levels of five AAs in 213 subjects with the risk of developing diabetes in an average of ten years. We further conducted a cross-sectional comparison of the serum levels of five AAs among 216 individuals with metabolically healthy or unhealthy status.

## Results

### The five AAs are predictive of the risk of future diabetes

**Figure 1:**
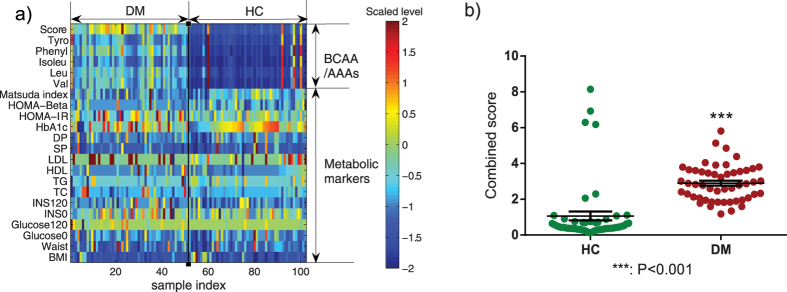
Heat map of AA and metabolic marker levels (**a**) and scatter plot of combined score (**b**) in individuals of healthy control (HC) and diabetes (DM) from longitudinal study. Abbreviations used: Score, the first decomposed principal component derived from the abundance of the five AAs; DM, future diabetes; DP, diastolic blood pressure; Glucose0, fasting glucose; Glucose120, 2 h glucose; HC, future healthy controls; HDL, high-density lipoprotein-cholesterol; HOMA-Beta = 20*INS0/(Glucose0-3.5); HOMA-IR = Glucose0*Glucose120/22.5; INS0, fasting insulin; INS120, 2 h insulin; Isoleu, isoleucine; LDL, low-density lipoprotein-cholesterol; Leu, leucine; Matsuda index = 10000/(Glucose0*Glucose120*INS0*INS120)1/2; Phenyl, phenylalanine; SP, systolic blood pressure; TC, total cholesterol; TG, triglyceride; Tyro, tyrosine; Val, valine.

The metabolic markers as well as the five AAs in the 51 future diabetes individuals (named DM) and 51 matched healthy individuals (named HC) were examined. There were no apparent differences in the metabolic markers between DM and HC groups at baseline (Table 1 and supplemental Table 1). However, the baseline serum levels of the five AAs were significantly increased in the DM group with fold changes higher than 2 and P values lower than 0.001 (Table 1). The heat map also showed larger variations in AAs between these two groups, compared to the metabolic markers (Fig. 1a). The inter-group variations and significance of the five AAs were similar to each other and the combined score was no better than their individuals (Table 1 and Fig. 1b).

We fitted basic and advanced logistic regression models adjusting for 3 and 14 confounding factors respectively, and further confirmed that these five AAs were strongly associated with diabetes risk and independent of both physical and key metabolic markers (ORs per s.d. > 2 and P values <=0.001, Table 1). However, the baseline metabolic markers were not able to predict the risk of diabetes (P values > 0.05, Table 1). The AUC analysis also demonstrated that the five AAs were better discriminators between these two groups (AUCs > 0.8, Table 1).

We regrouped the study participants with the same 51 individuals of future diabetes and 162 more heterogeneous healthy controls (supplemental Table 2). As expected, the five AAs and their combined score were significantly elevated in the DM group (FCs > 2, P values < 0.001, and AUCs > 0.8) and were strongly associated with future diabetes incidents (ORs per s.d. > 1.5 and P values  < 0.001 for both basic and advanced logistic models), outperforming any of the existing clinical markers. To summarize, the baseline serum levels of five AAs or their combined score showed superior capability in predicting the future development of diabetes over conventional metabolic markers.

### The five AAs were differentially expressed in healthy lean, overweight/obesity and diabetes individuals.

**Figure 2:**
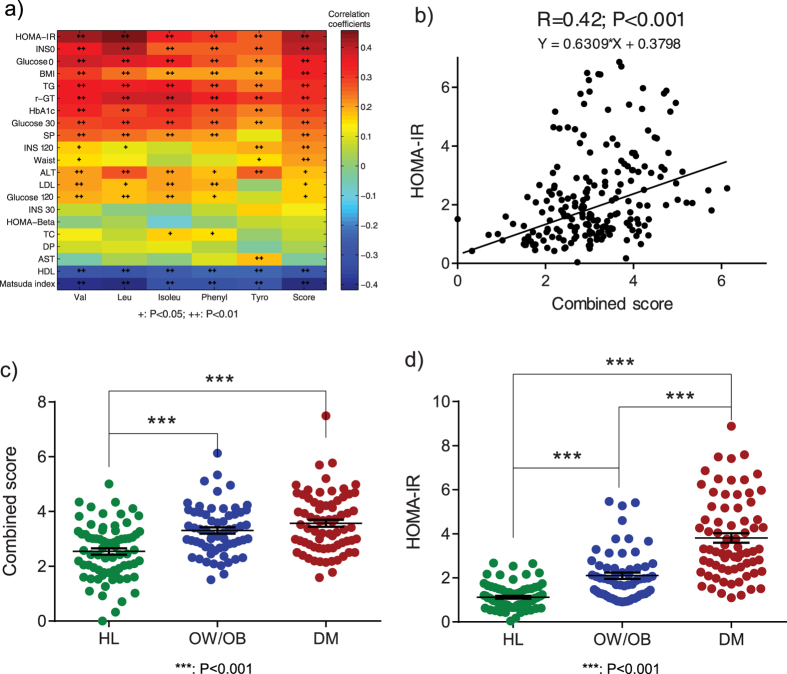
Heat map of Spearman correlation coefficients between AAs and metabolic markers (**a**), relationship between combined score and HOMA-IR (**b**), Scatter plot of combined score (**c**) and HOMA-IR (**d**) in HL, OW/OB, and DM individuals from cross sectional study. Abbreviations used: ALT, alanine aminotransferase; AST, aspartate aminotransferase; Score, the first decomposed principal component derived from the abundance of the five AAs; DM, overweight or obesity with type 2 diabetes; DP, diastolic blood pressure; γ-GT, gamma-glutamyl trans-supeptidase; Glucose0, fasting glucose; Glucose120, 2 h glucose; HDL, high-density lipoprotein-cholesterol; HL, healthy lean; HOMA-Beta = 20*INS0/(Glucose0-3.5); HOMA-IR = Glucose0*Glucose120/22.5; INS0, fasting insulin; INS120, 2 h insulin; Isoleu, isoleucine; LDL, low-density lipoprotein-cholesterol; Leu, leucine; Matsuda index = 10000/(Glucose0*Glucose120*INS0*INS120)1/2; OW/OB, healthy overweight or obesity; Phenyl, phenylalanine; SP, systolic blood pressure; TC, total cholesterol; TG, triglyceride; Tyro, tyrosine; Val, valine.

In the nested cross sectional study, 216 gender matched individuals were selected from the SHOS Study, including 72 healthy leans (HL), 72 healthy overweight or obese subjects (OW/OB), and 72 overweight or obese subjects diagnosed with type 2 diabetes (DM). We first assessed the correlations between five AAs and metabolic markers. The heat map showed their strong correlations (Fig. 2a). In particular, five AAs had the closest positive correlation with HOMA-IR, and the combined score reached the highest correlation (r = 0.42 and P < 0.001) (Fig. 2b). Their metabolic status discrimination performances were also examined and summarized in Table 2 and supplemental Table 3. The OW/OB group had significantly higher levels of the five AAs compared to the HL group (fold change range 1.07–1.19). Although not statistically different from OW/OB group, DM group showed even higher levels of AAs compared to HL (fold change range 1.11–1.30). The combined score of the five AAs (Fig. 2c), had the lowest p-values, highest fold changes, and largest AUC areas. Compared to the five AAs, some of the metabolic markers, particularly, the HOMA-IR, performed better in discriminating the three groups (Fig. 2d). In summary, five AAs were closely associated with metabolic status of individuals, especially insulin resistance, but not as good as some of the metabolic markers in distinguishing individuals with diabetes from their healthy counterparts.

## Discussion

In this work, we conducted two nested studies using participants (n = 429) from the SHDS and SHOS cohort studies to evaluate the performances of five AAs (valine, leucine, isoleucine, phenylalanine, and tyrosine) in predicting the risk of developing diabetes, and to understand the associations between the AAs and the metabolic status (e.g., insulin resistance) in Chinese populations. First, from the longitudinal study, the baseline serum levels of five AAs were significantly elevated in those who were diagnosed with diabetes 10 years later. Among the five AAs, valine stands out with a 251% increased odds (per s.d.) of developing diabetes in the future (P < 0.001, Table 1). Our results highlighted the predictive value of the five AAs for future diabetes, which supports previous studies conducted by Wang^9^, Weurtz^11^, and Batch^8^. Second, in the cross sectional comparison, the five AAs were progressively increased in overweight/obese and diabetes subjects, and closely associated with metabolic markers of insulin resistance (HOMA-IR), BMI, TG, HbA1c, etc. These findings are consistent with several studies on obese nondiabetic individuals^10^, metabolic abnormalities^8^, and in the middle-aged Finnish individuals^7^.

The performance of five circulating AAs over clinical metabolic makers in predicting or discriminating diabetes was different in two studies. In the longitudinal study the five AAs and their combined score outperformed clinical metabolic markers in diabetes risk prediction. This suggests that the blood levels of AAs elevated long before the rise of clinical markers in response to the onset of diabetes. In the second study, five AAs were differentially expressed in three groups, lean, obese, and obese with diabetes, with an increasing trend (i.e., HL < OW/OB < DM). However, they were not as strong markers as clinical metabolic markers such as HOMA-IR in distinguishing diabetic patients from their healthy counterparts.

These essential amino acids, valine, leucine, isoleucine, and phenylalanine, and a conditionally essential amino acid, tyrosine, play important roles in the synthesis of specific neurotransmitters, protein degradation and turnover, lymphocyte growth and proliferation, dendritic cell maturation, glycogen synthesis, energy metabolism, and so on^17^,^18^,^19^,^20^,^21^,^22^. They were reported as early indicators of cardiovascular^23^,^24^,^25^, pancreatic adenocarcinoma^26^, kidney disease^27^, and cardioembolic stroke^28^. Although not completely understood, there exists several possible mechanism underlying the association between these AAs and the risk of developing diabetes. First, type 2 diabetes begins with insulin resistance of peripheral tissues^29^,^30^. To compensate for this resistance, pancreatic beta-cells respond with increased insulin synthesis and proliferation^31^. It is estimated that the islet function may be reduced by up to 50%, compared with healthy control subjects, at the onset of diabetes^32^. Mammalian target of rapamycin complex 1 (mTORC1) and its downstream effectors such as S6 kinase-1 (S6K1) are important for growth and proliferation of beta cell as well as insulin secretion. Multiple amino acids, particularly leucine, are important regulators of mTORC1 signaling^33^. Elevated levels of plasma BCAAs for a long period may contribute to the hyperactivation of mTOR signaling and presumably result in early beta cell dysfunction and destruction^34^,^35^. Second, some observations suggested that BCAAs facilitate glucose uptake by liver and skeletal muscle as well as enhancing glycogen synthesis by insulin-independent manner through phosphatidylinositol 3-kinase (PI3-kinase) or protein kinase C (PKC) pathways rather than mTOR pathway^22^,^36^. Third, accumulating evidences suggest that intestinal microbiota composition and perturbation represent a critical environmental factor to the progression of diabetes^37^,^38^,^39^. Many bacterial species are involved in the synthesis of BCAA and decomposition of AAAs^40^. Therefore, altered microbiome and the association with AAs might be another possible mechanism of diabetes-related AA alterations. A fourth possibility is suggested by recent studies on the interplay of adipose tissue, BCAA metabolism, and glucose homeostasis^13^. The increased BCAAs may generate more catabolic intermediates propionyl CoA and succinyl CoA, leading to accumulation of incompletely oxidized fatty acids and glucose, mitochondrial stress, impaired insulin action, and ultimate perturbation of glucose homeostasis. Our recent study also demonstrated that circulating fatty acid levels were significantly elevated in pre-diabetic subjects compared to their healthy counterparts, and several unsaturated fatty acids were closely associated with the risk of future diabetes^41^. Finally, individual AAs performed differently when predicting future diabetes among various populations. Phenylalanine and valine were superior to the other 3 AAs in the studies with American populations^9^, tyrosine showed better performance among participants in South Asian^14^, whereas valine stood out in our study with Chinese population. In comparison, most diabetic patients in China had lower BMI, but with more abdominal fat^15^, which were presumably associated with earlier beta-cell dysfunction^42^ and specific genetic loci for diabetes^43^,^44^.

Our recent metabolomics study suggests that there are significant gender differences in metabolic profiles of obese individuals^16^. In this study, we compared all the data in male and female participants separately and found that the variations of the five AAs were consistent but slightly different between males and females, with more significant increases observed in male participants (supplemental Tables 4–7).

Key strengths of the present investigation are the use of two separate cohorts, with participants at several stages of diabetes development. The roles of five AAs were evaluated comprehensively by case-control samples and carefully selected longitudinal samples, and in male and female subjects, respectively. Comparing with a complete panel of clinical markers measured for all the participants, the five AAs showed a similar diagnostic power for diabetes but a remarkably improved predictive performance on future diabetes, highlighting their predictive value in clinical and epidemiological applications.

Several limitations in our study exist. First, we are well aware that statistical power would be increased with a larger sample size. The use of medium sample sizes in our study is primarily due to the strict inclusion criteria. Second, although fasting serum samples were used for analysis, the information regarding diet, sedentary lifestyle, and other possible confounders was not available for investigation.

## Methods

### Study populations

#### Longitudinal study

A group of 213 healthy individuals (20–75 year-old) was selected from a prospective cohort study called Shanghai Diabetes Study (SHDS)^45^. The SHDS started in 1997–2001, where baseline fasting serum of all the participants were collected and stored. After a median follow-up time of 10.0 years (SD = 3.1), 51 individuals (47% male) developed diabetes and 162 (27% male) remained free of diabetes or MS in accordance with WHO 1999 criteria and the proposed standard by the Chinese Diabetes Society (CDS)^46^,^47^. This group was analyzed in two different ways. The first way was to analyze 102 subjects in total, including 51 subjects who developed diabetes in 10 years and 51 matched individuals who remained metabolically healthy in 10 years. The second way was to analyze the same 51 future diabetes individuals and all of the 162 healthy subjects (213 subjects in total).

#### Cross-sectional study

A total of 216 Chinese adult individuals (20–65 year-old) including healthy lean, healthy overweight or obese, and overweight or obese with type 2 diabetes were selected from the Shanghai Obesity Study (SHOS)^48^. There were 72 participants, 36 males and 36 females, in each group. Participants with type 2 diabetes were newly diagnosed ones without any complications and antidiabetic medications. The selection of individuals into the healthy lean group and healthy overweight or obese group was based on the following 4 criteria: (1) lean means 18.5 kg/m^2^ ≤ BMI < 24.0 kg/m^2^, overweight means 24.0 kg/m^2^ ≤ BMI < 28.0 kg/m^2^, and obese means BMI ≥ 28 kg/m^2^ 49; (2) fasting glucose concentration ≤ 6.1 mmol/L and no previous history of diabetes; (3) systolic / diastolic blood pressure <140/90 mmHg and no previous high blood pressure history; and (4) fasting plasma TG < 1.7 mmol/L and fasting plasma HDL ≥ 0.9 mmol/L (men) or ≥1.0 mmol/L (women) and no previous history of high cholesterol. Individuals in both healthy lean group and healthy overweight or obese group were excluded if they had chronic inflammatory disease, cardiopulmonary, renal or liver disease, active malignancy, or were taking any medication (including weight loss or psychotropic medication).

### Sample collection

Serum samples of the two studies were collected and stored following the standard operation protocol of Sixth People’s Hospital of Shanghai, China. Briefly, fasting venous blood samples were centrifuged immediately after collection from the subjects in the morning, and the resulting serum were delivered by dry ice storage boxes to the laboratory study and stored in aliquots in a −80 °C freezer until sample preparation.

All the methods were carried out in accordance with the approved guidelines. All the studies were conducted with ethical approval from the sixth people’s hospital of Shanghai, China. Written informed consent was obtained from participants prior to inclusion in the two cohort studies, SHDS and SHOS, respectively.

### Metabolic markers

Fasting and 2 h postprandial plasma glucose and insulin levels, serum lipid profiles (total cholesterol TC, triglyceride TG, high-density lipoprotein-cholesterol HDL, low-density lipoprotein-cholesterol LDL), aspartate aminotransferase (AST), alanine aminotransferase (ALT), gamma-glutamyl trans-supeptidase (γ-GT), blood pressure (systolic and diastolic blood pressure SP and DP), waist, body mass index (BMI), and liver and kidney functions were determined as previously described^50^. Insulin resistance, secretion, and sensitivity was measured by HOMA-IR(Glucose0*Glucose120/22.5)^51^, HOMA-beta(20*INS0/(Glucose0-3.5))^51^, and Matsuda index(10000/(Glucose0*Glucose120*INS0*INS120))^1/2^ 52 calculated from the glucose and insulin levels. All study measures were obtained before 10 a.m. after an overnight fast in accordance as well with the standard operation protocol of the Sixth People’s Hospital of Shanghai, China.

### Measurement of BCAAs and AAAs

The serum levels of the five AAs in all the enrolled participants were analysed by ultra-performance liquid chromatography triple quadruple mass spectrometry (UPLC-TQ/MS, Waters, Milford, MA, USA). A 40 μL aliquot of serum sample was used in UPLC-TQ/MS ESI+ analysis. After diluted with 80 μL of water, the sample was extracted with 500 μL of a mixture of methanol and acetonitrile (1:9, v/v). The extraction procedure was performed at −20 °C for 10 min after 2 min vortexing and 1 min ultrasonication. The sample was then centrifuged at 4 °C at 12000 rpm for 15 min. An aliquot of 20 μL supernatant was vacuum-dried at room temperature. After that, the residue was redissolved by 100 μL of a mixture of methanol and water (1:1, v/v) with 1 μg/mL of L-2-chlorophenylalanine followed by the same vortexing, ultrasonication and centrifugation steps ahead. A volume of 80 μL supernatant was trasferred into the sampling vial for UPLC-TQ/MS analysis (Waters, Manchester, U.K.). In addition to the internal standards used for quality contol, a quality control (QC) samples consisting of five reference standards was prepared and run after each 10 serum samples. The QC samples were kept at 10 °C during the entire analysis. A 5 μL aliquot of sample was injected into an ultraperformance liquid chromatography system (Waters, U.K.) with a 4.6 mm × 150 mm, 5 μm Elispse XDB-C18 column (Angilent, USA). The column was held at 40 °C. The elution procedure for the column was 1% for the first 0.5 min,1–20% B over 0.5–9 min, 20–75% B over 9–11 min, 75–99% B over 11–16 min, and the composition was held at 99% B for 0.5 min, where A = water with 0.1% formic acid and B = acetonitrile with 0.1% formic acid for positive mode (ESI+) and the flow rate was 0.4 mL/min. A Waters XEVO-Triple Quadrupole MS was used for the mass spectrometry detection. The temperature for the source and desolvation gas was set at 150 and 450 °C respectively. The gas flow for cone and desolvation was 50 and 800 L/h respectively. The capillary voltage was set to 3.0 kV. All the compounds were detected in multiple reaction monitoring (MRM) mode.

### Statistics

The acquired raw data from UPLC-TQ/MS was targeted and processed by TargetLynx software (v 4.1, Waters, USA). Both internal standard (L-2-chlorophenylalanine) and quality control samples (mixture of standards) were used for sample pretreatment in order to ensure data quality and eliminate the run order effects of instrument detection. There were no missing values in the data set. A combined score was generated by the Factor Analysis based on the abundance of the five AAs (i.e., the first decomposed principal component explaining the largest data variance was taken as the combined score). Data in figures and tables were expressed as mean ± SEM. All statistical tests were two-sided, and p values less than 0.05 were considered statistically significant. All statistical analysis and graphics were carried out using SPSS (V19, IBM, USA), graphpad Prism (6.0, Graphpad, USA), and matlab (2014a, MathWorks, USA).

Mann Whitney U test was applied to examine the significance of the five AAs, the combined score, and metabolic markers as over 90% variables are deviated from normality from Kolmogorov-Smirnov normality test. Multiple testing corrections were not conducted considering that samples were matched, and the variable number was relatively small compared to the total number of samples. The ROC analysis was performed and area under ROC (AUC) was used to rank their diagnostic capabilities. The heat map profiling was based on z-score scaled data of metabolic markers or AAs. Spearman’s rank correlation coefficients were calculated to examine the associations between AAs/the combined score and metabolic markers. Both basic and advanced logistic regression models were constructed, based on SD scaled data, to assess the correlations of AAs with the risk of future diabetes. Basic logistic regression models were adjusted with confounding factors of baseline age, gender, and BMI. Advanced models were adjusted with the same three covariants of basic models and 11 more metabolic markers, including fasting and postprandial glucose, fasting and postprandial insulin, TC, TG, HDL, LDL, SP, DP, and HOMA-IR. Both basic and advanced models were fitted for five AAs and only basic models were fitted for metabolic markers.

## Additional Information

**How to cite this article**: Chen, T. *et al.* Branched-chain and aromatic amino acid profiles and diabetes risk in Chinese populations. *Sci. Rep.* **6**, 20594; doi: 10.1038/srep20594 (2016).

## Supplementary Material

Supplementary Information

## Figures and Tables

**Table 1:** Metabolic markers and AAs at baseline and their statistical significance in discriminating individuals who developed diabetes in 10 years (DM, n = 51) from those who remained metabolically healthy (HC, n = 51).

Metabolic markers and AAs	P1	FC	Basic/advanced logistic model		
			OR (95% CI)	P2	AUC
BMI (kg/m^2^)	0.05	0.97	0.98 (0.85, 1.13)	0.79	0.50
Waist (cm)	0.45	1.01	1.06 (0.99, 1.13)	0.11	0.58
Glucose0 (mM)	0.57	1.02	1.35 (0.57, 3.17)	0.50	0.55
Glucose120 (mM)	0.28	0.98	0.80 (0.53, 1.23)	0.31	0.53
INS0 (U/L)	0.53	0.99	0.98 (0.88, 1.09)	0.75	0.60
INS120 (U/L)	0.31	0.95	1.00 (0.98, 1.00)	0.60	0.55
TC (mM)	0.47	0.97	0.15 (0.02, 1.37)	0.09	0.54
TG (mM)	0.37	1.09	2.57 (0.54, 12.17)	0.23	0.56
HDL (mM)	0.06	0.97	0.22 (0.01, 5.66)	0.36	0.61
LDL (mM)	0.81	1.06	1.63 (0.67, 3.92)	0.28	0.55
SP (mmHg)	0.05	1.03	1.03 (0.98, 1.07)	0.30	0.60
DP (mmHg)	0.37	1.03	1.04 (0.97, 1.11)	0.26	0.55
HbA1c (%)	0.10	1.01	1.45 (0.44, 4.79)	0.55	0.57
HOMA-IR	0.71	1.01	0.94 (0.60, 1.48)	0.79	0.55
HOMA-Beta	0.69	1.09	1.00 (1.00, 1.00)	0.49	0.51
Matsuda index	0.54	0.85	1.00 (0.99, 1.00)	0.21	0.54
Valine	<0.001	2.52	3.04 (1.89, 4.87)/3.51 (1.75, 7.04)	<0.001/<0.001	0.93
Leucine	<0.001	2.06	2.08 (1.40, 3.09)/2.38 (1.50, 3.76)	<0.001/<0.001	0.88
Isoleucine	<0.001	2.60	2.61 (1.71, 4.00)/2.52 (1.58, 4.04)	<0.001/<0.001	0.89
Phenylalanine	<0.001	2.01	2.07 (1.38, 3.09)/2.22 (1.40, 3.50)	<0.001/ 0.001	0.89
Tyrosine	<0.001	2.28	3.03 (1.86, 4.94)/2.96 (1.70, 5.14)	<0.001/<0.001	0.89
Combined score	<0.001	2.72	2.53 (1.68, 3.81)/2.55 (1.61, 4.03)	<0.001/<0.001	0.91

Abbreviations used: AUC, area under ROC curve; CI, confidence interval; Combined score: the first decomposed principal component derived from the abundance of the five AAs; DP, diastolic blood pressure; FC, fold change; Glucose0, fasting glucose; Glucose120, 2h glucose; HDL, high-density lipoprotein-cholesterol; HOMA-Beta = 20*INS0/(Glucose0-3.5); HOMA-IR = Glucose0*Glucose120/22.5; INS0, fasting insulin; INS120, 2h insulin; LDL, low-density lipoprotein-cholesterol; Matsuda index = 10000/(Glucose0*Glucose120*INS0*INS120)^1^^/^2; OR, Odds ratio; SP, systolic blood pressure; TC, total cholesterol; TG, triglyceride.

P1 were from Mann Whitney U test.

FC represent mean ratio of DM to HC.

Odds ratio (OR) and confidence interval (CI) per s.d., and P (P2) values were from basic and advanced logistical regression models and S.D. scaled data.

**Table 2:** Statistical significance of metabolic markers and AAs in discriminating individuals of healthy lean (HL, n = 72), healthy overweight or obese (OW/OB, n = 72), and overweight or obese with diabetes (DM, n = 72).

Metabolic markers and AAs	P			FC			AUC		
	HL vs OW/OB	HL vs DM	OW/OB vs DM	OW/OB /HL	DM/HL	DM/ OW/OB	HL vs OW/OB	HL vs DM	OW/OB vs DM
BMI (kg/m2)	<0.001	<0.001	0.35	1.33	1.35	1.02	1.00	1.00	0.55
Waist (cm)	<0.001	<0.001	<0.001	1.14	1.07	0.93	0.97	0.77	0.80
TC (mM)	0.40	<0.001	<0.001	0.99	1.25	1.26	0.54	0.84	0.86
TG (mM)	0.05	<0.001	<0.001	1.16	2.80	2.41	0.60	0.93	0.88
HDL (mM)	<0.001	<0.001	<0.01	0.86	0.77	0.89	0.70	0.83	0.64
LDL (mM)	<0.01	<0.001	<0.001	1.08	1.31	1.21	0.64	0.79	0.72
γ-GT (U/L)	0.72	<0.001	<0.001	1.17	1.78	1.51	0.52	0.84	0.76
SP (mmHg)	0.82	<0.001	<0.001	1.01	1.17	1.16	0.52	0.82	0.80
DP (mmHg)	0.13	<0.001	<0.001	1.02	1.12	1.09	0.58	0.73	0.68
ALT (U/L)	0.10	<0.001	0.05	1.08	1.31	1.22	0.58	0.66	0.60
AST (U/L)	0.79	0.53	0.69	0.99	0.99	1.00	0.52	0.54	0.52
HbA1c (%)	0.01	<0.001	<0.001	1.03	1.60	1.55	0.62	0.96	0.95
Glucose 0 (mM)	0.58	<0.001	<0.001	1.02	1.58	1.56	0.53	0.91	0.92
Glucose 30 (mM)	0.91	<0.001	<0.001	1.00	2.59	2.60	0.51	0.99	1.00
Glucose 120 (mM)	0.50	<0.001	<0.001	0.99	1.33	1.33	0.54	1.00	0.99
INS0 (U/L)	<0.001	<0.001	0.01	1.82	2.17	1.20	0.81	0.89	0.63
INS30 (U/L)	<0.001	0.01	<0.001	1.57	0.87	0.55	0.72	0.62	0.79
INS120 (U/L)	0.06	<0.001	<0.001	1.24	2.43	1.96	0.59	0.82	0.76
HOMA-IR	<0.001	<0.001	<0.001	1.87	3.40	1.82	0.80	0.96	0.81
HOMA-beta	<0.001	0.18	<0.001	1.71	0.90	0.53	0.76	0.57	0.79
Matsuda index	<0.001	<0.001	<0.001	0.57	0.27	0.48	0.76	0.99	0.85
Valine	<0.001	<0.001	0.07	1.18	1.26	1.07	0.69	0.74	0.59
Leucine	<0.01	<0.001	0.07	1.19	1.30	1.09	0.65	0.72	0.59
Isoleucine	0.04	<0.001	0.06	1.13	1.22	1.08	0.61	0.69	0.59
Phenylalanine	<0.01	<0.001	0.16	1.15	1.21	1.05	0.65	0.70	0.57
Tyrosine	0.04	<0.01	0.45	1.07	1.11	1.04	0.60	0.64	0.54
Combined score	<0.001	<0.001	0.18	1.30	1.40	1.08	0.71	0.76	0.57

Abbreviations used: ALT, alanine aminotransferase; AST, aspartate aminotransferase; AUC, area under ROC curve; Combined score: the first decomposed principal component derived from the abundance of the five AAs; DP, diastolic blood pressure; FC, fold change; γ-GT, gamma-glutamyl trans-supeptidase; Glucose0, fasting glucose; Glucose120, 2h glucose; HDL, high-density lipoprotein-cholesterol; HOMA-Beta = 20*INS0/(Glucose0-3.5); HOMA-IR = Glucose0*Glucose120/22.5; INS0, fasting insulin; INS120, 2h insulin; LDL, low-density lipoprotein-cholesterol; Matsuda index = 10000/(Glucose0*Glucose120*INS0*INS120)^1/2^; SP, systolic blood pressure; TC, total cholesterol; TG, triglyceride.

P values were from Mann Whitney U test.
