# Supplementary material for: Branched-chain and aromatic amino acid profiles and diabetes risk in Chinese populations
Source: Sci Rep. 2016 Feb 5;6:20594. doi: 10.1038/srep20594 (PMC4742847; doi:10.1038/srep20594)
Supplement: Supplementary Information [file srep20594-s1.pdf]

Branched-chain and aromatic amino acid profiles and diabetes risk in Chinese populations

Supplementary Information

Tianlu Chen[1,#], Yan Ni[2, #], Xiaojing Ma[3], Yuqian Bao[1,3], Jiajian Liu[1], Fengjie Huang[1], Cheng Hu[1,3], Guoxiang Xie[2], Aihua Zhao[1], Weiping Jia[1,3*], Wei Jia[1,2*]

[1]Shanghai Key Laboratory of Diabetes Mellitus and Center for Translational Medicine, Shanghai Jiao Tong University Affiliated Sixth People's Hospital, Shanghai 200233, China.

[2]University of Hawaii Cancer Center, Honolulu, HI 96813, USA.

[3]Department of Endocrinology and Metabolism, Shanghai Jiao Tong University Affiliated Sixth People's Hospital; Shanghai Diabetes Institute; Shanghai, 200233, China.

# These authors contributed equally to this work.

*Correspondence should be addressed to W.P.J. (wpjia@sjtu.edu.cn) or W.J. (wjia@cc.hawaii.edu).

Abbreviations:

Glucose0, fasting glucose;

Glucose120, 2h glucose;

INS0, fasting insulin;

INS120, 2h insulin;

TC, total cholesterol;

TG, triglyceride.

γ-GT, gamma-glutamyl trans-supeptidase;

SP, systolic blood pressure;

DP, diastolic blood pressure;

HDL, high-density lipoprotein-cholesterol;

LDL, low-density lipoprotein-cholesterol;

ALT, alanine aminotransferase;

AST, aspartate aminotransferase;

HOMA-Beta=20*INS0/(Glucose0-3.5);

HOMA-IR= Glucose0*Glucose120/22.5;

Matsuda index=10000/(Glucose0*Glucose120*INS0*INS120)$^{1/2}$;

Combined score, the first decomposed principal component derived from the abundance of the five AAs;

FC, fold change;

AUC, area under ROC curve;

OR, Odds ratio;

CI, confidence interval;

Data were presented as mean ± SEM.

SI Table 1 Baseline metabolic markers of longitudinal study.

Metabolic markers	All healthy controls (n=162)	Matched healthy controls (n=51)	Future diabetes (n=51)
Gender (male:female)	44:118	24:27	24:27
Age (yrs)	38.51±0.95	41.02±2.01	53.78±1.75
BMI (kg/m2)	24.63±0.27	26.40±0.45	25.53±0.50
Waist (cm)	76.91±0.71	79.14±1.20	80.12±1.13
Glucose0 (mM)	4.76±0.04	5.03±0.05	5.13±0.09
Glucose120 (mM)	5.21±0.09	6.18±0.13	6.08±0.18
INS0 (U/L)	7.90±0.40	9.43±0.69	9.36±0.56
INS120 (U/L)	45.15±3.46	59.67±6.57	56.42±4.20
TC (mM)	4.05±0.03	4.08±0.05	3.97±0.02
TG (mM)	1.05±0.02	1.23±0.03	1.33±0.06
HDL (mM)	1.36±0.01	1.37±0.02	1.33±0.02
LDL (mM)	2.66±0.03	2.73±0.04	2.88±0.10
SP (mmHg)	112.16±0.93	118.56±1.29	122.51±1.51
DP (mmHg)	73.41±0.54	77.16±0.84	79.14±1.13
HbA1c (%)	5.70±0.01	5.73±0.02	5.80±0.07

SI Table 2 Metabolic markers and AAs at baseline and their statistical significance in discriminating individuals who developed diabetes in 10 years (DM, n=51) from those who remained metabolically healthy (HC, n=162).

Metabolic markers and AAs	P1	FC	Basic/advanced logistic model		AUC
			OR (95% CI)	P2	
BMI(kg/m2)	0.22	0.96	1.04 (0.93, 1.17)	0.50	0.58
Waist(cm)	0.05	1.01	1.00 (0.95, 1.06)	0.94	0.60
Glucose0(mM)	<0.001	1.02	3.06 (1.49, 6.29)	<0.01	0.67
Glucose120(mM)	<0.001	0.98	1.46 (1.05, 2.02)	0.02	0.68
INS0(U/L)	<0.001	1.18	1.04 (0.96, 1.12)	0.53	0.65
INS120(U/L)	<0.001	1.25	1.01 (1.00, 1.01)	0.32	0.71
TC(mM)	0.38	0.97	0.31 (0.08, 1.16)	0.08	0.55
TG(mM)	<0.001	1.09	8.55 (2.12, 34.39)	<0.01	0.74
HDL(mM)	0.42	0.96	0.67 (0.07, 6.89)	0.74	0.55
LDL(mM)	0.42	1.06	1.82 (0.82, 4.02)	0.14	0.58
SP (mmHg)	<0.001	1.05	1.05 (1.01, 1.09)	0.01	0.73
DP (mmHg)	<0.001	1.03	1.10 (1.03, 1.16)	<0.01	0.72
HbA1c (%)	<0.01	1.01	2.79 (0.64, 12.18)	0.17	0.58
HOMA-IR	<0.001	1.29	1.24 (0.92, 1.69)	0.16	0.68
HOMA-Beta	0.84	0.93	1.00 (1.00, 1.00)	0.46	0.51
Matsuda index	<0.001	0.53	0.99 (0.98, 1.00)	<0.01	0.75
Valine	<0.001	2.52	1.83 (1.51, 2.23)/2.48 (1.66, 3.72)	<0.001/<0.001	0.91
Leucine	<0.001	2.06	1.94 (1.37, 2.76)/1.67 (1.29, 2.16)	<0.001/<0.001	0.87
Isoleucine	<0.001	2.60	1.50 (1.27, 1.83)/1.88 (1.39, 2.52)	<0.001/<0.001	0.86
Phenylalanine	<0.001	2.01	1.42 (1.18, 1.71)/1.74 (1.31, 2.32)	<0.001/<0.001	0.86
Tyrosine	<0.001	2.28	1.50 (1.24, 1.81)/1.87 (1.39, 2.52)	<0.001/<0.001	0.86
Combined score	<0.001	2.72	1.47 (1.25, 1.75)/1.82 (1.38, 2.38)	<0.001/<0.001	0.88

P1 values were from Mann Whitney U test.

FC represent mean ratio of DM to HC.

Odds ratio (OR) and confidence interval (CI) per s.d., and P2 values were from basic and advanced logistical regression model, based on S.D. scaled data.

SI Table 3 Metabolic markers of cross sectional study.

Metabolic markers	Healthy lean (n=72)	Overweight or obese (n=72)	Overweight or obese with diabetes (n=72)
Gender (male:female)	36:36	36:36	36:36
Age (yrs)	52.35±0.76	51.29±0.69	56.91±0.83
BMI (kg/m2)	20.48±0.07	27.26±0.25	27.73±0.30
Waist (cm)	86.72±0.45	99.08±0.72	92.45±0.83
TC (mM)	4.36±0.07	4.32±0.06	5.44±0.11
TG (mM)	0.86±0.03	0.99±0.05	2.40±0.17
HDL (mM)	1.64±0.04	1.42±0.04	1.27±0.03
LDL (mM)	2.50±0.05	2.71±0.05	3.28±0.09
γ-GT (U/L)	21.07±1.12	24.70±2.23	37.42±2.54
SP (mmHg)	116.50±1.28	117.34±1.41	136.66±2.59
DP (mmHg)	73.11±0.86	74.61±1.08	81.61±1.60
ALT (U/L)	17.56±0.91	18.95±0.92	23.04±1.24
AST (U/L)	21.10±0.53	20.95±0.58	20.90±0.69
Glucose0 (mM)	5.05±0.05	5.20±0.05	8.08±0.31
Glucose 30 (mM)	8.52±0.22	8.66±0.20	13.50±0.42
Glucose 120 (mM)	5.74±0.14	5.72±0.13	14.87±0.51
HbA1c (%)	5.42±0.03	5.39±0.06	7.18±0.16
INS0 (U/L)	4.95±0.28	9.00±0.58	10.77±0.53
INS30 (U/L)	45.45±2.97	67.54±4.82	37.30±2.96
INS120 (U/L)	27.81±2.03	34.51±2.91	67.64±5.52

SI Table 4 Metabolic markers and AAs at baseline and their statistical significance in discriminating male individuals who developed diabetes in 10 years (DM, n=24) from those who remained metabolically healthy (HC, n=24).

Metabolic markers and AAs	P1	FC	OR (95% CI)	P2	AUC
BMI(kg/m2)	0.74	0.99	1.01 (0.81, 1.26)	0.96	0.55
Waist(cm)	0.27	0.98	0.98 (0.89, 1.08)	0.68	0.58
Glucose0(mM)	0.72	1.02	1.29 (0.37, 4.46)	0.69	0.54
Glucose120(mM)	0.94	1.00	0.93 (0.53, 1.62)	0.79	0.53
INS0(U/L)	0.83	1.11	1.04 (0.89, 1.21)	0.63	0.55
INS120(U/L)	0.17	1.17	1.00 (0.99, 1.02)	0.65	0.60
TC(mM)	0.95	0.99	3.74 (0.17, 84.88)	0.41	0.51
TG(mM)	0.12	1.06	5.22 (0.25, 107.29)	0.28	0.63
HDL(mM)	0.13	0.95	0.08 (0.00, 17.27)	0.35	0.64
LDL(mM)	0.84	1.06	1.92 (0.58, 6.35)	0.28	0.55
SP (mmHg)	0.30	1.01	1.00 (0.93, 1.07)	0.89	0.58
DP (mmHg)	0.94	1.01	1.05 (0.95, 1.17)	0.36	0.57
HbA1c (%)	0.05	1.02	1.21 (0.33, 4.45)	0.77	0.57
HOMA-IR	0.46	1.13	1.18 (0.61, 2.28)	0.63	0.61
HOMA-Beta	0.65	1.28	1.00 (1.00, 1.01)	0.24	0.55
Metsuda index	0.31	0.70	0.99 (0.99, 1.00)	0.15	0.61
Valine	<0.001	3.06	5.49 (1.57, 19.27)	<0.01	0.96
Leucine	<0.001	2.52	2.92 (1.42, 6.00)	<0.01	0.94
Isoleucine	<0.001	3.71	4.70 (1.97, 11.20)	<0.001	0.95
Phenylalanine	<0.001	2.56	3.47 (1.50, 8.04)	<0.01	0.96
Tyrosine	<0.001	2.78	4.84 (1.96, 11.95)	<0.01	0.94

P1 values were from Mann Whitney U test.

FC represent mean ratio of DM to HC.

Odds ratio (OR) and confidence interval (CI) per s.d., and P2 values were from basic logistical regression model, based on S.D. scaled data.

SI Table 5 Metabolic markers and AAs at baseline and their statistical significance in discriminating female individuals who developed diabetes in 10 years (DM, n=27) from those who remained metabolically healthy (HC, n=27).

Metabolic markers and AAs	P1	FC	OR (95% CI)	P2	AUC
BMI(kg/m2)	0.03	0.95	0.96 (0.79, 1.15)	0.64	0.62
Waist(cm)	0.99	1.04	1.14 (1.03, 1.26)	0.01	0.52
Glucose0(mM)	0.63	1.02	1.39 (0.41, 4.68)	0.60	0.52
Glucose120(mM)	0.14	0.97	0.70 (0.38, 1.29)	0.25	0.75
INS0(U/L)	0.41	0.91	0.94 (0.82, 1.08)	0.38	0.53
INS120(U/L)	0.97	0.79	0.99 (0.96, 1.01)	0.21	0.51
TC(mM)	0.30	0.96	0.02 (0.00, 1.05)	0.05	0.57
TG(mM)	0.88	1.12	2.06 (0.36, 11.72)	0.42	0.50
HDL(mM)	0.23	0.98	0.44 (0.01, 24.54)	0.69	0.60
LDL(mM)	0.56	1.06	1.42 (0.35, 5.71)	0.62	0.56
SP (mmHg)	0.10	1.05	1.06 (0.98, 1.13)	0.13	0.60
DP (mmHg)	0.27	1.04	1.04 (0.95, 1.13)	0.38	0.53
HbA1c (%)	0.70	1.01	2.69 (0.22, 33.42)	0.44	0.57
HOMA-IR	0.96	0.93	0.79 (0.45, 1.41)	0.43	0.51
HOMA-Beta	0.36	0.95	1.00 (0.99, 1.01)	0.65	0.52
Metsuda index	0.86	1.08	1.01 (0.99 1.03)	0.41	0.56
Valine	<0.001	2.12	2.50 (1.48, 4.23)	<0.01	0.88
Leucine	<0.001	1.70	1.70 (1.06, 2.73)	0.03	0.83
Isoleucine	<0.001	1.96	1.94 (1.19, 3.16)	<0.01	0.84
Phenylalanine	<0.001	1.64	1.67 (1.05, 2.65)	0.03	0.84
Tyrosine	<0.001	1.95	2.31 (1.32, 4.04)	<0.01	0.84

P1 values were from Mann Whitney U test.

FC represent mean ratio of DM to HC.

Odds ratio (OR) and confidence interval (CI) per s.d., and P2 values were from basic logistical regression model, based on S.D. scaled data.

SI Table 6 Statistical significance of metabolic markers and AAs in discriminating male individuals of healthy lean (HL, n=36), healthy overweight or obese (OW/OB, n=36), and overweight or obese with diabetes (DM, n=36).

Metabolic markers and AAs	P			FC			AUC		
	HL vs OW/OB	HL vs DM	OW/OB vs DM	OW/OB /HL	DM/HL	DM/ OW/OB	HL vs OW/OB	HL vs DM	OW/OB vs DM
BMI (kg/m2)	<0.001	<0.001	0.96	1.34	1.35	1.01	1.00	1.00	0.50
Waist (cm)	<0.001	<0.001	<0.01	1.16	1.10	0.95	0.99	0.87	0.72
TC (mM)	0.32	<0.001	<0.001	0.97	1.28	1.32	0.57	0.86	0.89
TG (mM)	0.01	<0.001	<0.001	1.29	2.82	2.18	0.69	0.91	0.85
HDL (mM)	<0.001	<0.001	0.13	0.80	0.76	0.95	0.81	0.87	0.61
LDL (mM)	0.02	<0.001	<0.001	1.11	1.38	1.25	0.69	0.85	0.77
γ-GT (U/L)	<0.01	<0.001	<0.01	1.42	2.04	1.43	0.74	0.88	0.71
SP (mmHg)	0.55	<0.001	<0.001	1.02	1.19	1.17	0.56	0.87	0.81
DP (mmHg)	0.13	<0.01	0.10	1.03	1.10	1.07	0.63	0.69	0.62
ALT (U/L)	0.34	0.03	0.15	1.07	1.23	1.15	0.56	0.64	0.61
AST (U/L)	0.07	0.19	0.63	0.91	0.94	1.03	0.65	0.60	0.54
HbA1c (%)	0.49	<0.001	<0.001	1.05	1.61	1.54	0.67	0.95	0.93
Glucose0 (mM)	0.02	<0.001	<0.001	1.00	1.52	1.51	0.51	0.91	0.94
Glucose 30 (mM)	0.91	<0.001	<0.001	0.94	2.65	2.81	0.58	1.00	1.00
Glucose 120 (mM)	0.37	<0.001	<0.001	1.01	1.34	1.33	0.56	0.99	0.98
INS0 (U/L)	<0.001	<0.001	0.57	2.17	2.33	1.07	0.87	0.90	0.54
INS30 (U/L)	<0.001	0.05	<0.001	2.10	0.91	0.44	0.81	0.64	0.85
INS120 (U/L)	0.13	<0.001	<0.01	1.41	2.78	1.98	0.61	0.83	0.74
HOMA-IR	<0.001	<0.001	<0.001	2.23	3.59	1.61	0.86	0.97	0.76
HOMA-beta	<0.001	0.51	<0.001	2.13	0.99	0.46	0.78	0.55	0.78
Matsuda index	<0.001	<0.001	<0.001	0.47	0.25	0.53	0.81	0.99	0.78
Valine	<0.001	<0.001	0.89	1.27	1.27	1.00	0.78	0.75	0.51
Leucine	0.01	<0.01	0.74	1.24	1.26	1.01	0.70	0.72	0.53
Isoleucine	0.02	<0.01	0.77	1.17	1.18	1.01	0.68	0.70	0.52
Phenylalanine	0.01	<0.001	0.17	1.19	1.26	1.06	0.68	0.76	0.60
Tyrosine	0.02	0.03	0.80	1.13	1.13	1.00	0.68	0.65	0.52

P values were from Mann Whitney U test.

FC represent mean ratio of OW/OB to HL, DM to HL, and DM to OW/OB.

SI Table 7 Statistical significance of metabolic markers and AAs in discriminating female individuals of healthy lean (HL, n=36), healthy overweight or obese (OW/OB, n=36), and overweight or obese with diabetes (DM, n=36).

Metabolic markers and AAs	P			FC			AUC		
	HL vs OW/OB	HL vs DM	OW/OB vs DM	HL vs OW/OB	HL vs DM	OW/OB vs DM	HL vs OW/OB	HL vs DM	OW/OB vs DM
BMI(kg/m2)	<0.001	<0.001	0.20	1.32	1.35	1.02	1.00	1.00	0.59
Waist(cm)	<0.001	0.02	<0.001	1.13	1.03	0.91	0.95	0.66	0.88
TC(mM)	0.52	<0.001	<0.001	0.99	1.21	1.22	0.54	0.81	0.83
TG(mM)	0.64	<0.001	<0.001	1.07	2.77	2.59	0.53	0.95	0.92
HDL(mM)	0.02	<0.001	0.01	0.90	0.78	0.88	0.66	0.82	0.67
LDL(mM)	0.19	<0.001	<0.01	1.06	1.25	1.18	0.59	0.74	0.69
γ-GT(U/L)	0.22	<0.001	<0.001	1.00	1.48	1.48	0.58	0.82	0.83
SP (mmHg)	0.74	<0.001	<0.001	1.00	1.16	1.16	0.52	0.76	0.77
DP (mmHg)	0.39	<0.001	<0.01	1.02	1.13	1.11	0.56	0.77	0.72
ALT (U/L)	0.07	<0.01	0.40	1.15	1.43	1.24	0.62	0.69	0.56
AST (U/L)	0.17	0.75	0.35	1.08	1.05	0.97	0.59	0.52	0.56
HbA1c(%)	0.75	<0.001	<0.001	1.02	1.59	1.56	0.58	0.97	0.96
Glucose0(mM)	0.26	<0.001	<0.001	1.05	1.66	1.58	0.56	0.93	0.89
Glucose 30(mM)	0.41	<0.001	<0.001	1.03	2.53	2.47	0.54	0.99	0.99
Glucose 120(mM)	0.53	<0.001	<0.001	0.98	1.31	1.34	0.52	1.00	0.99
INS0(U/L)	<0.001	<0.001	<0.01	1.57	2.05	1.31	0.75	0.89	0.70
INS30(U/L)	0.03	0.15	<0.001	1.19	0.82	0.69	0.65	0.60	0.74
INS120(U/L)	0.39	<0.001	<0.001	1.10	2.19	1.99	0.56	0.85	0.82
HOMA-IR	<0.001	<0.001	<0.001	1.61	3.24	2.01	0.75	0.95	0.84
HOMA-beta	<0.001	0.20	<0.001	1.42	0.83	0.59	0.73	0.59	0.81
Matsuda index	<0.01	<0.001	<0.001	0.69	0.30	0.43	0.69	0.99	0.90
Valine	0.05	<0.001	0.03	1.13	1.26	1.12	0.63	0.74	0.65
Leucine	0.06	<0.01	0.17	1.19	1.35	1.13	0.63	0.70	0.59
Isoleucine	0.27	<0.01	0.08	1.13	1.27	1.13	0.57	0.68	0.62
Phenylalanine	0.08	0.04	0.73	1.13	1.16	1.03	0.62	0.64	0.52
Tyrosine	0.48	0.07	0.27	1.02	1.09	1.07	0.55	0.62	0.57

P values were from Mann Whitney U test.

FC represent mean ratio of OW/OB to HL, DM to HL, and DM to OW/OB.

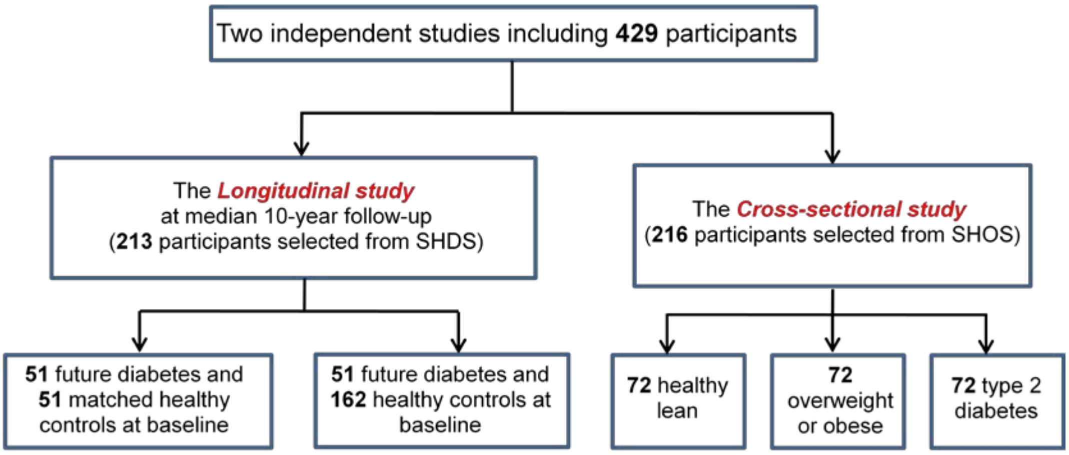

Notes: SHOS, Shanghai Obesity Study;
SHDS, Shanghai Diabetes Study

SI Figure1 Participant flow chart
